# The complete mitochondrial genome of *Paramesotriton chinensis* (Caudata: Salamandridae) and phylogenetic studies of *Paramesotriton*

**DOI:** 10.1080/23802359.2017.1325344

**Published:** 2017-05-19

**Authors:** Jia Yang, Lipeng Yu, Shuyan Zhang, Rilin Liu, Cangsong Chen, Hung-Du Lin

**Affiliations:** a Zhejiang Museum of Natural History, Hangzhou, Zhejiang, China;; b The Administration Bureau of Longwangshan Natural Reserve, Anji, Zhejiang, China;; c The Administration Bureau of Qingliangfeng Natural Reserve, Linan, Zhejiang, China;; d The Administration Bureau of Dayanghu Natural Reserve, Jinning, Zhejiang, China;; e The Affiliated School of National Tainan First Senior High School, Tainan, Taiwan

**Keywords:** Mitogenome, phylogeny, *Paramesotriton chinensis*

## Abstract

The complete mitochondrial genome of the Asian warty newt *Paramesotriton chinensis* was sequenced. The complete mitogenome of *P. chinensis* is a circular double-stranded DNA sequence that is 16,361 bp long and was biased toward A + T content at 61.3% (33.0% A, 28.3% T, 23.9% C, and 14.7% G). The complete mitogenome of *P. chinensis* consists of 13 protein-coding genes (PCGs), 22 transfer RNAs (tRNAs), 1 ribosomal RNAs (16S rRNA), and 1 putative control region. This study presented the complete mitogenome of *P. chinensis* and provided essential and important DNA molecular data for further phylogenetic and evolutionary analysis for genus *Paramesotriton*.

The salamandrid genus *Paramesotriton*, currently consists of 14 nominal species endemic to southern China, except for *P. deloustali* which is mainly distributed in northern Vietnam. This genus commonly known as the Asian warty newts, consists of two species groups, which have been recovered as reciprocally monophyletic sister groups in molecular phylogenetic analyses (Gu et al. 2012; Yuan et al. 2014, 2016). Recent studies indicate several investigators who established different phylogenetic relationships for *Paramesotriton* based on different molecular markers (Weisrock et al. 2006; Gu et al. 2012; Yuan et al. 2014, 2016). The advent of molecular studies and the increasing number of genes and DNA regions used in the phylogenetic studies led to structuring of the taxonomy of *Paramesotriton*, improved understanding of the phylogenetic relationships of species. *Paramesotriton chinensis* is distributed in Zhejiang and Jiangxi Province, China. In this study, we propose to determine the complete mitochondrial genome using next-generation sequencing (NGS), which may verify the phylogenetic position of this species.

Specimen of *P. chinensis* was obtained from Zhejiang and deposited at the Zhejiang Museum of Natural History, Hangzhou, Zhejiang, China. Genomic DNA extraction and next-generation sequencing were described in previous publication (Shen et al. 2016). Initially, the raw next generation sequencing reads generated from HiSeq 2000 (Illumina, San Diego, CA) were altered to remove low-quality reads. Around 0.06% raw reads (7944 out of 13,849,558) were subjected to *de novo* assembly using commercial software (Geneious V9, Auckland, New Zealand) to produce a single, circular form of complete mitogenome with about an average of 122 × coverage. The mitogenomic sequence has been deposited into GenBank under the accession number KY609177.

The complete mitogenome of *P. chinensis* is a circular double-stranded DNA sequence that is 16,361 bp long and was biased toward A + T content at 61.3% (33.0% A, 28.3% T, 23.9% C, and 14.7% G). DOGMA (Wyman et al. 2004), ARWEN (Laslett & Canback 2008), and MITOS (Bernt et al. 2013) programs were used to predict the protein-coding, rRNA and tRNA genes of *P. chinensis* mitogenome. The complete mitochondrial genome of *P. chinensis* had the same gene arrangement and similar compositions according with other *Paramesotriton* species.

To further explore its taxonomic status and the phylogenetic relationship within *Paramesotriton*, we used MEGA6 software (Tamura et al. 2013) to construct a Maximum likelihood tree (with 500 bootstrap replicates) containing complete mitogenomes of seven species. These seven *Paramesotriton* species are recovered as a monophyletic group strongly supported, and could be divided into clades A (*P. hongkongensis*, *P. chinensis*, *P. deloustali,* and *P. guangxiensis*) and clade B (*P. caudopunctatus*, *P. laoensis* and *P. longliensis*) (Figure 1). Our molecular data also reveal a close phylogenetic relationship among *P. chinensis*, *P. deloustali*, and *P. guangxiensis*, and this result was supported by previous phylogenetic studies (Gu et al. 2012; Yuan et al. 2014). This study presented the complete mitogenome of *P. chinensis* and provided essential and important DNA molecular data for further phylogenetic and evolutionary analysis for genus *Paramesotriton*.

**Figure 1.**
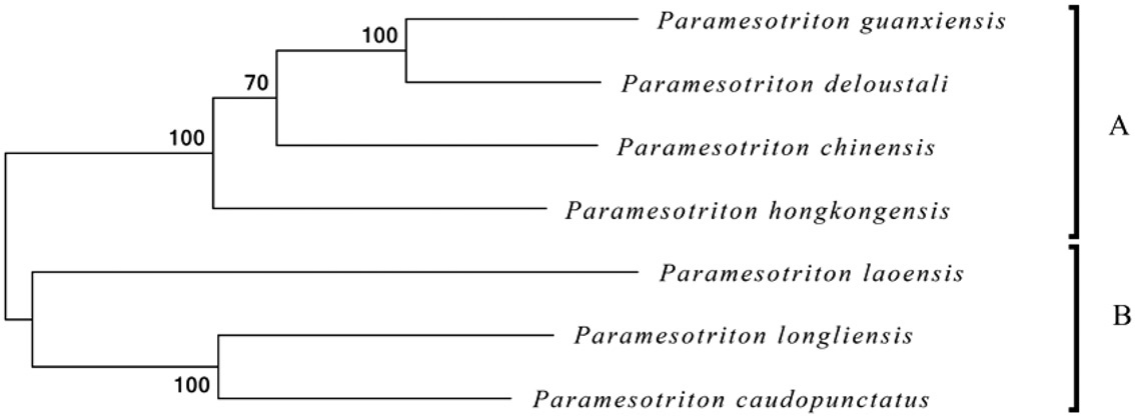
Maximum likelihood (ML) phylogenetic tree based on the complete mitochondrial genome sequences of 6 closely related *Paramesotriton* species Numbers at the branches indicate bootstrapping values with 500 replications. Accession number: *Paramesotriton longliensis* (NC032310), *Paramesotriton caudopunctatus* (EU880326), *Paramesotriton laoensis* (EU880328), *Paramesotriton guanxiensis* (NC032309), *Paramesotriton deloustali* (EU880327), *Paramesotriton hongkongensis* (NC006407).
